# Greenhouse Gas Fluxes from Salt Marshes Exposed to Chronic Nutrient Enrichment

**DOI:** 10.1371/journal.pone.0149937

**Published:** 2016-02-25

**Authors:** Gail L. Chmura, Lisa Kellman, Lee van Ardenne, Glenn R. Guntenspergen

**Affiliations:** 1 Department of Geography, McGill University, Montreal, Quebec, Canada; 2 Environmental Sciences Research Centre, St. Francis Xavier University, Antigonish, Nova Scotia, Canada; 3 United States Geological Survey, Patuxent Wildlife Research Center, Laurel, Maryland, United States of America; MESC; University of South Alabama, UNITED STATES

## Abstract

We assessed the impact of nutrient additions on greenhouse gas fluxes using dark static chambers in a microtidal and a macrotidal marsh along the coast of New Brunswick, Canada approximately monthly over a year. Both were experimentally fertilized for six years with varying levels of N and P. For unfertilized, N and NPK treatments, average yearly CO_2_ emissions (which represent only respiration) at the microtidal marsh (13, 19, and 28 mmoles CO_2_ m^-2^ hr^-1^, respectively) were higher than at the macrotidal marsh (12, 15, and 19 mmoles m^-2^ hr^-1^, respectively, with a flux under the additional high N/low P treatment of 21 mmoles m^-2^ hr^-1^). Response of CH_4_ to fertilization was more variable. At the macrotidal marsh average yearly fluxes were 1.29, 1.26, and 0.77 μmol CH_4_ m^-2^ hr^-1^ with control, N, and NPK treatments, respectively and 1.21 μmol m^-2^ hr^-1^ under high N/low P treatment. At the microtidal marsh CH_4_ fluxes were 0.23, 0.16, and -0.24 μmol CH_4_ m^-2^ hr^-1^ in control, N, and NPK and treatments, respectively. Fertilization changed soils from sinks to sources of N_2_O. Average yearly N_2_O fluxes at the macrotidal marsh were -0.07, 0.08, and 1.70, μmol N_2_O m^-2^ hr^-1^ in control, N, NPK and treatments, respectively and 0.35 μmol m^-2^ hr^-1^ under high N/low P treatment. For the control, N, and NPK treatments at the microtidal marsh N_2_O fluxes were -0.05, 0.30, and 0.52 μmol N_2_O m^-2^ hr^-1^, respectively. Our results indicate that N_2_O fluxes are likely to vary with the source of pollutant nutrients but emissions will be lower if N is not accompanied by an adequate supply of P (e.g., atmospheric deposition vs sewage or agricultural runoff). With chronic fertilization the global warming potential of the increased N_2_O emissions may be enough to offset the global cooling potential of the C sequestered by salt marshes.

## Introduction

Documentation of greenhouse gas fluxes in wetlands is needed to assess the role these ecosystems play in global climate change (e.g., [1]). Salt marsh soils, in particular, have been shown to be important sinks for carbon dioxide, CO_2_ [2]. On an aerial basis, saltmarsh soils store more carbon (C) than forests, and retain it over millennia [3]. The C stored in salt marshes has recently been branded as “blue carbon” in conjunction with efforts to develop standards for verifying their potential for mitigating global climate change so that they can be placed on the carbon market [3].

In the absence of nutrient enrichment, salt marshes appear to have negligible emissions of the more potent greenhouse gases nitrous oxide, N_2_O (e.g., [4–5]) and methane, CH_4_ [5]. These two gases have global warming potentials 298 and 34 times greater, respectively than CO_2_ [6]. Nitrous oxide can be released from fertilized terrestrial soils [7] and CH_4_ is released from freshwater wetlands [5]. Some studies have shown that fertilization can increase the emissions of greenhouse gases in salt marshes, thus reducing their values as C sinks, but the research available on response of salt marshes to nutrient enrichment has been limited.

Although salt marshes are not considered to be sources of N_2_O [4,8] or CH_4_ [5] under unimpacted conditions, field studies with experimentally fertilized salt marshes suggest that those with an adequate supply of nitrogen (N) may become sources of N_2_O [4] and CH_4_ [9]. Mosemann-Valtiera et al. [4] measured significant emissions of N_2_O immediately after fertilization of a Massachusetts marsh with nitrate (NO_3_^-^) solutions. The limited fertilization changed it, for a short time, from a sink to a source of this greenhouse gas. Tobias et al. [10] subjected a Virginia salt marsh to a high NO_3_^-^ load and determined that 50–60% was released as N_2_O. Irvine et al. [9] fertilized a California salt marsh for several months with a slow release granular urea and found the CH_4_ flux increased linearly with the level of N applied. Irvine et al. [9] surmised that N stimulated CH_4_ production in salt marsh soils. Additionally, meta-analysis of CH_4_ flux in non-wetland soils revealed that high levels of N amendments had an inhibitory effect on oxidation of CH_4_ in upper soil layers of that produced in lower layers [11].

Experiments suggest that increased supply of N to salt marshes through coastal cultural eutrophication (due to runoff, sewage and wet deposition of nitrate from the atmosphere) could cause increased N_2_O and CH_4_ emissions. However, coastal cultural eutrophication is the result of long-term addition of N to waters and soils and different pollution sources will provide varying proportions of other nutrients. For instance, atmospheric deposition provides N as NO_3_^-^ while agricultural runoff and sewage may provide N as NO_3_^-^ or NH_4_^+^, in combination with other nutrients, particularly phosphorus (P). This differences could be significant because the microbial community responsible for N transformations is P limited [12] and the long-term N additions from cultural eutrophication could result in a shift in the N:P ratio of soils, thus the response of the microbial community. The meta-analysis of CH_4_ uptake in non-wetland soils by Aronson and Helliker [11] indicates that the historical soil N status and type of applied fertilizer will affect the CH_4_ response. Thus, it is possible that previous research on the response of N_2_O and CH_4_ fluxes to fertilization of salt marsh soils, which has been limited to less than seven months and fertilization with application limited to N may not fully reveal how the greenhouse gas flux from salt marshes can respond to cultural eutrophication.

Variation in environmental conditions within salt marshes may further affect the flux of CH_4_ and N_2_O in response to N addition, thus were measured in our study. Some environmental factors stimulate production of CH_4_, such as reducing conditions (indicated by high water tables) or lack of sulfate (indicated by salinity). (Reduction of sulfate is energetically preferential to the reduction of CO_2_ performed by methanogens.) Both CH_4_ and N_2_O fluxes may be affected by variations in temperature [9, 13–14]. In their review Childers et al. [15] conclude that export of dissolved nutrients from marsh soils increases with tidal range. Thus, if microtidal marshes retain more reactive N than macrotidal ones we assume the former would have a greater response in N_2_O emissions with N additions, a point we consider in our study.

In our study we examine whether chronic fertilization results in higher emissions of N_2_O or CH_4_ within a microtidal and macrotidal marsh, if emissions vary with different proportions of N:P in fertilizer applications, and if they do, what this means in terms of climate feedbacks. To accomplish this, we collected data over a full annual period from two long-term fertilization experiments in salt marshes on the coast of New Brunswick. Since microbial activity is affected by environmental factors such as water table, temperature, and salinity, we also examine whether variation in these factors are predictors of the flux of N_2_O or CH_4_.

## Methods

### Study Areas

**Fig 1:**
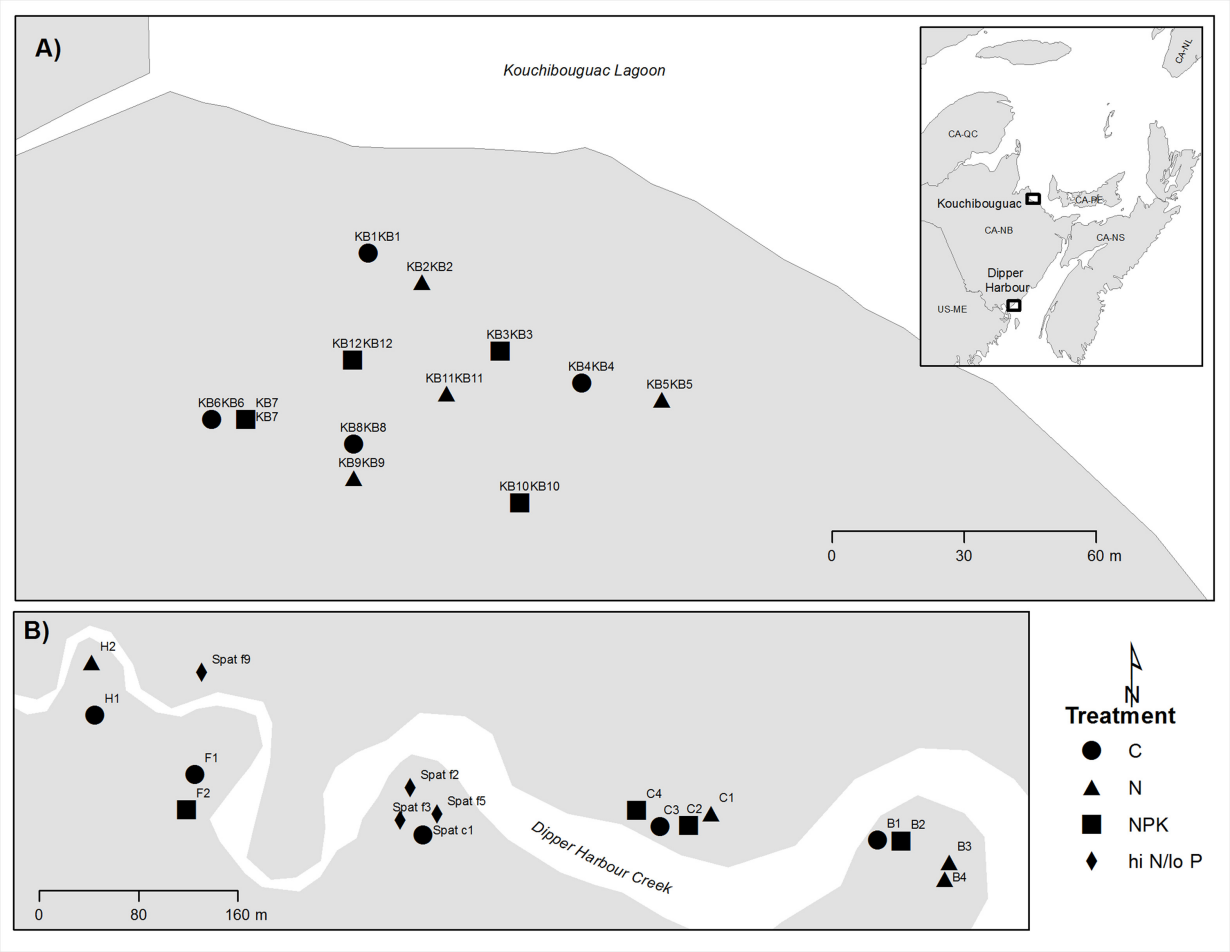
Schematic map of gas flux sample locations. (a) Sample locations at the Kouchibouguac marsh. (b) Sample locations at the Dipper Harbour marsh. Each symbol represents a chamber location. Inset shows location of both sites on the New Brunswick coast. C = control, N = fertilizer with only nitrogen applied, NPK = fertilizer with nitrogen, phosphorus and potassium applied, hi N/lo P = fertilizer with high levels of nitrogen.

Our study was conducted in two salt marshes on the coast of New Brunswick, Canada (Fig 1). Dipper Harbour marsh (45°05' N, 66°26' W), previously described by Daoust et al. [16] and [17], is located in the macrotidal Bay of Fundy and has a tidal amplitude of ~6 m. Kouchibouguac marsh (46° 46' N, 64° 54' W), which corresponds to the site of core 2 described by Chmura et al. [8], is located on the microtidal (sensu [18]) Gulf of St. Lawrence and has a tidal amplitude of 1 m. Both locations have a semidiurnal tidal cycle [19–20].

Permits were obtained for research at both sites. Work at Kouchibouguac marsh, located at Kouchibouguac National Park was carried out with a permit from Parks Canada. Work at Dipper Harbour was carried out with permission of land owner New Brunswick Power and with a permit from the Province of New Brunswick Department of Environment and Local Government.

### Field Sampling

Out study areas, Dipper Harbour and Kouchibouguac marshes are sites of long-term fertilization experiments. Gas flux samples were collected in areas of high marsh dominated by *Spartina patens* and our experimental design took advantage of two different long term projects that involved fertilization of *S*. *patens-*dominated vegetation plots. One project involved the application of Premium Vigoro® containing 16% N (1.57% ammonia N, 14.43% urea N); 4% P (as P_2_O_5_); and 8% K (as K_2_O) or application of Nutriform® (Nu-gro Technologies, Inc.) containing 38% N (5.0% urea N, 8.0% slowly available water soluble N, and 25% water insoluble N). Hereafter we refer to these treatments as NPK and N. Beginning in 2006 these applications were made yearly in spring and late summer, from which we assume a monthly loading rate of 5.5 g N m^-2^. The other fertilization project also was located in *S*. *patens*-dominated marsh, but only at Dipper Harbour. The project involved the application of Scotts® Turf Builder® containing 29% N (5.3% ammonia N, 13.1% urea N, 9.7% other water soluble N, and 0.9% water insoluble N); 3% P (as P_2_O_5_); and 4% K (as K_2_O). Beginning in 2004 these applications were made every 2–3 weeks from April to September, from which we assume a monthly loading rate of 24.9 g N m^-2^. Hereafter, this treatment will be referred to as “high N/low P”. We note that the high N/low P fertilizer provided an N:P in a ratio of ~8:1 while the ratio of the NPK treatment was much lower, 4:1.

Samples were collected approximately monthly from July 4, 2011 to July 2, 2012 during low tide. Four chambers were installed for each fertilizer treatment and control (for which locations had been randomly selected) as shown in Fig 1. Gas samples were collected through a dark static chamber technique described by Magenheimer et al. [21]. Chambers were covered in reflective material to prevent heating by sunlight. In July 2011 we used a 7.5-l chamber, but all following months used a 17-l chamber. The 25 cm diameter chambers were set into the rim of plastic collars which had been inserted into the soil weeks before the first sample event and collars were left in the soil for the duration of the study. Gas was collected through a syringe attached to the chamber with tygon tubing, once every 20 minutes for 1 hour (providing four samples to calculate flux). The 60 cc syringe was pumped to mix gases in the tubing with chamber gases before each sample was collected. Gas was transferred to 12 ml N_2_-purged and evacuated Exetainer vials.

Environmental measurements were taken outside of, but within 1 m of the chamber collar. Soil temperature over 15 cm depth was recorded while gas samples were being collected and average daily air temperatures were downloaded from Environment Canada (http://climate.weather.gc.ca/, last accessed Aug 15, 2014) for nearby meteorological stations at Kouchibouguac (46°46'11" N, 65°00'24" W) and Saint John (45°19'05" N, 65°53'08 " W). Water table depth was measured and porewater samples for salinity were collected after completion of gas sampling using methods described by Yu and Chmura [22]. The need to maintain conditions in plots through the fall and spring prevented us from harvesting vegetation to determine biomass. During the summer of 2011, we did, however, count the number of live grass stems within each collar.

### Laboratory Analyses

Gas samples were analyzed in the laboratory on a Varian GC450 equipped with an autosampler (CombiPal) and detectors for N_2_O (ECD), CH_4_ (FID) and CO_2_ (TCD) analyses. Calibration curves were established (Matheson Tri-Gas standards) for N_2_O (0.1, 1.0, 100 ppmv), CH_4_ (0, 10, 100 ppmv) and CO_2_ (100, 1000, 10000 ppmv). Gas standards were run as part of the analysis sequences to monitor instrument stability. Calculations of gas flux included changes in headspace concentration calculated based on the Ideal Gas Law and used the flux summation method described by McVicar and Kellman [23].

### Statistical Analyses

A one-way ANVOA analysis was performed to test for differences in mean gas flux between different treatments for both marshes. Student’s t-tests were used to determine if significant differences in stem density or gas flux between marshes in each treatment type were present. Data which differed significantly from a normal distribution were log-transformed. Nitrous oxide and CH_4_ flux from Dipper Harbour were transformed using the equations ln(N_2_O flux + 1.712) and ln(CH_4_ flux + 9.96), respectively. Only CO_2_ flux data from Kouchibouguac needed to be natural log transformed. Post hoc pair-wise comparisons to determine differences in treatments were performed using Tukey’s HSD when equal factor variance could be assumed (determined using Levene’s test) or Games-Howell when not, or when there was a large imbalance in factor group sizes.

Linear and best subsets multiple regression analysis was performed to determine if the observed differences in gas flux could be explained by differences in environmental variables in each marsh. Multiple regression selection criteria were set to provide the best overall adjusted R^2^ value. Non-significant predictor variables above the 5% level were then discarded until only significant variables remained. In the case of non-normal distributions flux data was transformed using natural logarithms to better approach normality. Kouchibouguac N_2_O data was transformed using the equation ln(flux + 1.447) and CH_4_ using ln(CH_4_ flux + 6.04). Dipper Harbour data was transformed in the same manner as described for the ANOVA analysis. Regression analysis was performed for all treatments individually. Analyses were performed using IBM SPSS 21.

## Results

### Environmental Conditions

**Fig 2:**
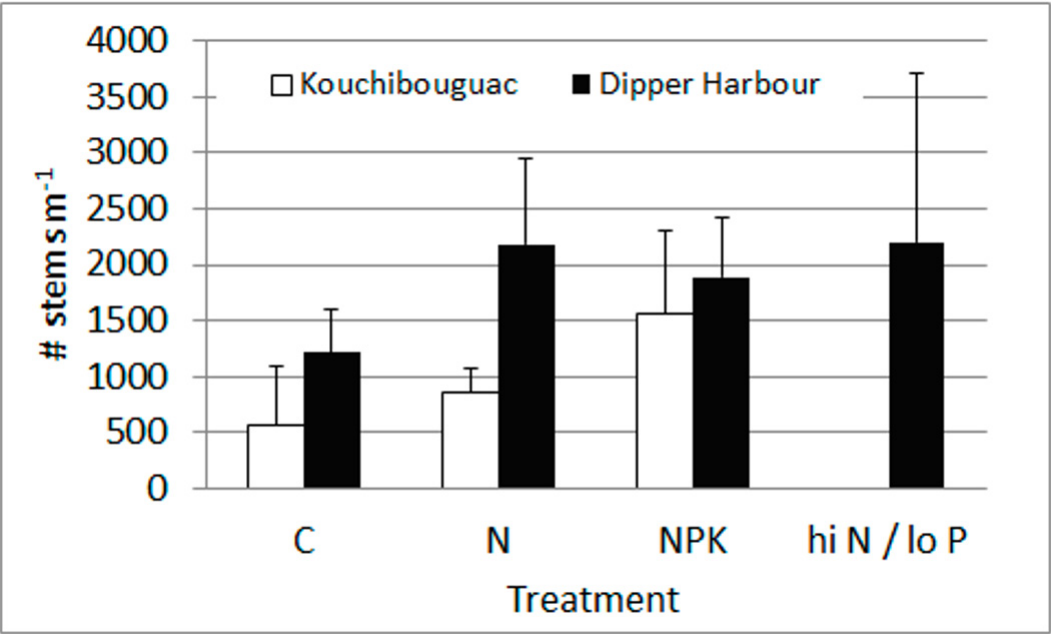
Average number of grass stems in plots by treatment. Error bars = ±1 sd. Treatment codes are explained in text and caption for Fig 1.

Our environmental measurements include soil and air temperature (Table 1), porewater salinity, water table depth (Table 2) and number of grass stems in each flux collar (Fig 2). The greatest range of both average daily air temperatures on sample days (-2.6 to 24.1°C) and soil temperatures (0.6 to 17°C) occurred at Kouchibouguac marsh. Average salinity of soil porewaters was highly variable within treatments and sample events, with the exception of Kouchibouguac porewater salinity (Table 2). Average porewater salinity was >18 during all sample events at Dipper Harbour, while salinities at Kouchibouguac were often lower. During sample events at both marshes water table depths were below the soil surface with the exception of some plots at Kouchibouguac in October and June (Table 2). At Dipper Harbour there was considerable variation in water table depth within treatments and sample events. The number of grass stems within flux collars was generally higher at Dipper Harbour than at Kouchibouguac, but the difference was only significant in the N alone treatment (t = -3.304, p = 0.016). At both marshes the average number of grass stems was higher with fertilization treatments, but these differences were not significant (Fig 2).

**Table 1:** Average (avg) and standard deviation (sd) of temperature (temp) measurements (°C) by treatment and sample event at Dipper Harbour and Kouchibouguac salt marshes.

	Control				N alone treatment				NPK treatment							
Kouchibouguac	air temp		soil temp		air temp		soil		air temp		soil temp					
Sample dates	avg	sd	avg	sd	avg	sd	avg	sd	avg	sd	avg	sd				
July 4, 2011	20.6	1	15.2	0.7	20.4	0.5	14.1	1.1	20.4	1.0	13.7	0.6				
Aug 8, 2011	18.8	0.1	16.4	1.1	18.7	0.1	17	0.8	18.7	0.2	16	0.4				
Sept 13, 2011	22.8	2.4	14.3	0.5	23.8	1.7	14.5	1.0	24.1	1.7	13.8	0.5				
Oct 7, 2011	6.7	0.8	7.4	0.8	6.7	0.8	7.6	1.7	7.2	0.7	6.5	0.4				
Nov 4, 2011	-2.6	0.6	5.8	1.3	-2.0	0.8	5.5	0.5	-2.5	0.9	7.2	1.2				
April 2, 2012	6.6	0.2	0.6	0.5	6.7	0.2	0.0	0.8	6.6	0.2	0.3	0.5				
May 12, 2012	12.2	1.2	4.4	0.8	12.5	0.9	4.1	0.3	12.9	1.2	4.4	0.5				
June 28, 2012	19.0	1.2	14.3	0.5	20.1	0.4	14.3	0.5	18.8	1.2	13.3	2.2				
	Control				N alone treatment				NPK treatment				hi N / lo P treatment			
Dipper Habour	air temp		soil temp		air temp		soil temp		air temp		soil temp		air temp		soil temp	
Sample dates	avg	sd	avg	sd	avg	sd	avg	sd	avg	sd	avg	sd	avg	sd	avg	sd
July 5, 2011	12.3	0.5	13.9	0.3	12.9	0	12.7	0.5	12.5	0.6	13	0.4	12.0	0.0	14.7	0.7
Aug 7, 2011	14.7	0.5	15.5	0.7	14.7	0.4	15.8	0.3	15.3	0.8	16.1	1.3	16.1	0.4	15.8	0.5
Sept 11, 2011	16.4	1.1	14.7	0.6	16.1	1.4	13	2.4	16.1	0.9	13.8	1	15.5	0.7	14	0.4
Oct 8, 2011	18.3	0.5	10.2	1.5	18.3	0.4	10.4	1.5	18.6	0.3	9.5	1.3	17.9	0.3	8.9	1.1
Nov 19 & 20, 2011	10.3	1.3	5.6	1.4	9.4	1.1	5.1	0.3	9.5	1.2	5.1	0.8	11.1	0.0	7.4	1.5
March 20, 2012	12.2	4.2	4.4	0.9	9.5	4.0	2.6	1.2	9.4	4.1	3.5	1.3	13.0	1.1	4.4	0.8
Jun 30-Jul 2, 2012	20.1	1.1	15.1	0.6	18.7	1.9	14.6	0.5	20.4	2.1	14.8	0.3	20.2	0	14.8	0.5

**Table 2:** Average (avg) and standard deviation (sd) of water table depth and salinity by treatment and sample event at Dipper Harbour and Kouchibouguac salt marshes. In April soil porewater was frozen, thus no measurements were available.

Dipper Harbour	water table depth (cm)		salinity		water table depth (cm)		salinity		water table depth (cm)		salinity		water table depth (cm)		salinity	
Sample dates	avg	sd	avg	sd	avg	sd	avg	sd	avg	sd	avg	sd	avg	sd	avg	sd
	Control				N				NPK				high N / low P			
July 5, 2011	3.2	3.6	21	8	8.2	6.3	19	10	0.5	0.4	21	3	1.8	2.0	25	1
Aug 7, 2011	6.1	7.2	26	4	3.4	3.2	28	5	3.3	1.2	25	7	4.1	3.0	30	1
Sept 11, 2011	5.3	3.8	24	4	4.9	3.0	24	9	3.6	3.8	25	4	4.1	2.1	28	1
Oct 8, 2011	8.5	3.5	24	5	11.4	3.7	24	9	9.5	4.1	24	4	10.8	2.3	26	3
Nov 19 & 20, 2011	6.0	2.9	21	5	6.9	4.6	20	7	4.4	2.1	21	4	8.2	3.0	27	1
March 20, 2012	6.2	5.3	23	4	6.2	6.3	22	8	4.7	2.8	22	4	10.6	4.1	24	5
Jun 30—Jul 2, 2012	3.5	2	27	5	5.3	1.1	24	11	2.9	2	25	8	3.5	2.2	30	10
Kouchibouguac	Control				N				NPK							
July 4, 2011	5.4	3.8	11	1	2.1	4.1	14	1	3.2	2.6	12	1				
Aug 8, 2011	-1.4	2.2	7	4			9	4		2.3	10	3				
Sept 13, 2011	15.0	0.0	10	1	13.7	2.6	10	2	15.0	0.0	10	1				
Oct 7, 2011	1.5	4.2	21	1	-2.6	0.2	22	1	-0.1	1.8	21	1				
Nov 4, 2011	7.6	6.0	18	1	1.4	2.4	18	3	3.7	1.7	20	0				
May 12, 2012	7.4	8.1	13	2	3.3	1.8	16	3	4.5	1.3	16	2				
June 28, 2012	4.0	5.3	16	3	8.2	11.5	22	2	-0.6	1.2	21	3				

Salinity is reported as Practical Salinity Units.

We used linear and multiple regressions to determine if soil temperature, air temperature, water table depth or grass stem density were significant predictors of CH_4_ or N_2_O fluxes. None of the environmental variables alone, or in combination (in a multiple regression) could significantly explain the CH_4_ variability. Nitrous oxide flux was only significantly related to stem density at Kouchibouguac marsh (r^2^ = 0.306, p = 0.005).

### Greenhouse Gas Fluxes with Fertilization

Monthly CO_2_ fluxes were generally higher with fertilization treatments, but highly variable (Table 3), thus statistically significant differences were not detected. Averaged over the study period, CO_2_ fluxes for Control, N and NPK treatments at Kouchibouguac (13, 19, and 28 mmoles CO_2_ m^-2^ hr^-1^, respectively) were higher than at Dipper Harbour (12, 15, 19 mmoles CO_2_ m^-2^ hr^-1^, respectively) with a flux under high N/low P treatment of 21 mmoles m^-2^ hr^-1^. However, t-tests showed that the difference between marshes is only significant with the NPK treatment (p = 0.012, Table 3).

**Table 3:** Average CO_2_ fluxes by sample event in μmoles m^-2^ hr^-1^.

Dipper Harbour	C			N			NPK			high N / low P		
date	average	sd	n	average	sd	n	average	sd	n	average	sd	n
5-Jul-11	13126	3120	4	17153	3403	3	20727	9828	4	27523	18794	3
09-Aug-11	22255	15508	5	15453	6267	3	17854	7068	4	38572	22140	4
12-Sep-11	11957	3411	5	17917	10849	4	12424	3102	4	15253	4645	4
08-Oct-11	9414	3710	5	19128	9318	4	21401	4996	4	17603	5316	4
19-Nov-11	2040	575	5	1922	982	4	2540	179	4	3823	563	4
20-Mar-12	4184	2434	5	4472	2998	4	4239	1360	4	7068	2851	4
Jun-July 2012	23001	7923	5	32294	10176	4	52385	18325	3	35628	10223	4
overall average	12,282			15,477			18,796			20,781		
Kouchibuguac												
4-Jul-11	21786	11916	4	27789	18510	4	44864	22077	4			
08-Aug-11	11955	5719	4	20028	11168	4	37203	8448	4			
13-Sep-11	19858	7741	4	25951	16885	4	46146	12075	4			
07-Oct-11	8779	6344	4	10186	1604	4	8439	3889	4			
20-Nov-11	2436	1035	4	5215	821	3	3531	2033	3			
Apr-12	3676	1741	4	2994	1651	4	3922	1126	4			
02-May-12	6077	989	4	6525	3257	4	7030	2222	4			
Jun-12	33000	7437	4	50214	30608	4	75341	34105	4			
overall average	13,446			18,613			28,310					

Fluxes of CH_4_ showed no significant differences with fertilization treatments, but there was a difference between marshes (Table 4). A t-test (p = 0.005) comparing CH_4_ fluxes averaged over all treatments and the entire sample period shows that fluxes were significantly higher at Dipper Harbour (1.09 μmol CH_4_ m^-2^ hr^-1^) than at Kouchibouguac (0.07 μmol CH_4_ m^-2^ hr^-1^).

**Table 4:** Average CH_4_ fluxes by sample event in μmoles m^-2^ hr^-1^.

Dipper Harbour	C			N			NPK			high N / low P		
date	avg	sd	n	avg	sd	n	avg	sd	n	avg	sd	n
05-Jul-11	2.90	7.13	4	1.95	3.10	3	-0.50	7.51	4	1.23	3.06	3
09-Aug-11	-0.54	1.24	5	1.64	0.30	3	0.54	1.03	4	1.84	2.40	4
12-Sep-11	1.93	5.46	5	1.80	4.28	4	1.76	1.69	4	1.16	1.35	4
08-Oct-11	0.57	1.20	5	0.04	0.62	4	0.07	1.05	4	2.33	1.52	4
19-Nov-11	0.90	3.19	5	0.78	1.41	4	1.63	1.40	4	1.58	1.75	4
20-Mar-12	1.2	1.68	5	0.62	1.21	4	1.44	1.35	4	-0.11	1.42	4
Jun-July 2012	2.01	5.26	5	2.02	4.72	4	0.42	0.83	4	0.42	2.74	4
overall	1.29			1.26			0.77			1.21		
Kouchibouguac												
04-Jul-11	0.80	0.58	4	0.39	1.26	4	-0.62	1.53	4			
08-Aug-11	0.02	0.49	4	0.89	0.91	4	0.79	1.97	4			
13-Sep-11	-0.07	1.37	4	0.07	0.73	4	0.53	1.08	4			
07-Oct-11	0.78	0.56	4	-1.15	1.82	4	-1.30	2.26	4			
20-Nov-11	0.15	3.50	4	-0.62	1.32	3	-1.90	1.38	3			
Apr-12	0.89	1.86	4	0.86	0.22	4	0.41	1.94	4			
02-May-12	-1.03	2.82	4	0.91	0.73	4	0.15	0.39	4			
29-Jun-12	0.32	1.97	4	-0.11	0.87	4	0.04	1.53	4			
overall	0.23			0.16			-0.24					

At both sites many of the monthly average N_2_O fluxes were low or negative (Table 5), yet we did detect a statistically significant effect of chronic fertilization. When considered over the entire sampling period at Kouchibouguac marsh, fluxes from plots fertilized with N (0.30 μmoles N_2_O m^-2^ hr^-1^, p<0.01) and NPK (0.52 μmoles N_2_O m^-2^ hr^-1^, p<0.001) were significantly higher than those from control plots which were a sink for N_2_O (-0.05 μmoles N_2_O m^-2^ hr^-1^). Over the study period, control plots at Dipper Harbour marsh were consistently a sink for N_2_O and averaged -0.1 μmoles N_2_O m^-2^ hr^-1^. Nitrous oxide fluxes from plots with the NPK treatment (1.70 μmoles N_2_O m^-2^ hr^-1^) were significantly higher than control plots (p = 0.001), plots fertilized with N-alone (0.08 μmoles N_2_O m^-2^ hr^-1^, p<0.005), and those fertilized with high N/low P fertilizer (0.35 μmoles N_2_O m^-2^ hr^-1^, p = 0.05). However, fluxes from plots fertilized with N-alone and high N/low P fertilizer were not significantly different from each other when considered over this period.

**Table 5:** Average N_2_O fluxes by sample event in μmoles m^-2^ hr^-1^.

Dipper Harbour	C			N			NPK			high N / low P		
date	avg	sd	n	avg	sd	n	avg	sd	n	avg	sd	
5-Jul-11	-0.10	0.20	4	0.11	0.07	3	0.53	0.17	4	2.62	0.13	3
09-Aug-11	-0.02	0.13	5	0.00	0.00	3	-0.04	0.07	4	0.14	0.20	4
12-Sep-11	-0.02	0.13	5	0.04	0.07	4	5.18	4.20	4	0.14	0.20	4
08-Oct-11	-0.14	0.14	5	0.04	0.24	4	4.12	0.82	4	-0.22	0.30	4
19-Nov-11	-0.01	0.12	5	-0.04	0.07	4	0.22	0.19	4	-0.11	0.14	4
20-Mar-12	-0.06	0.13	5	-0.00	0.17	4	0.09	0.11	4	0.00	0.17	4
June-July 2012	-0.11	0.34	5	0.39	0.29	4	1.81	1.14	4	-0.14	0.00	4
overall	-0.07			0.08			1.70			0.35		
Kouchibouguac												
Jul-11	-0.17	0.20	4	0.78	0.61	4	1.39	0.47	4			
08-Aug-11	0.00	0.00	4	-0.04	0.07	4	0.43	0.61	4			
13-Sep-11	0.04	0.18	4	0.04	0.07	4	0.63	0.73	4			
07-Oct-11	-0.04	0.14	4	-0.11	0.25	4	0.26	0.58	4			
20-Nov-11	0.00	0.18	4	0.00	0.15	3	0.15	0.153	3			
Apr-12	-0.04	0.08	4	0.07	0.09	4	0.11	0.14	4			
02-May-12	-0.11	0.07	4	0.91	0.73	4	0.07	0.19	4			
Jun-12	-0.07	0.14	4	0.75	1.02	4	1.07	0.86	4			
overall	-0.05			0.30			0.52					

There also was a difference in N_2_O fluxes with fertilization treatments between marshes. Under the N-alone treatment average N_2_O fluxes from Kouchibouguac were significantly higher (p = 0.002) than those from Dipper Harbour. In contrast, under the NPK treatment, average fluxes at Dipper Harbour were more than three times higher than at Kouchibouguac (p = 0.05). Under control conditions there was no difference between N_2_O fluxes of the two marshes.

## Discussion

Soils with chronic nutrient enrichment surprisingly had lower CH_4_ emissions as compared to controls. At Dipper Harbour the treatment with the highest proportion of P had the greatest decrease in CH_4_ flux and at Kouchibouguac, plots with the same NPK treatment, on average, became CH_4_ sinks. The decrease in CH_4_ emissions may be an indirect result of effects of plant growth. Plots with added fertilizer tended to have more robust growth, and presumably denser rhizospheres which would increase aeration of the soil and oxidation of CH_4_ (e.g., [24]). In contrast, in California salt marshes Irvine et al. (2012) showed increased CH_4_ emissions with addition of N at loading rates that exceeded ~10 g N m^-2^ yr^-1^. We note that the vegetation in the marsh studied by Irvine et al. [9] was dominated by *Salicornia* which has no rhizomes, thus the same effect may not be expected.

Plants also can shunt CH_4_ from sites of production in lower soil levels which have a lower redox potential. Methane fluxes were higher at Dipper Harbour than at Kouchibouguac marsh, although the latter has lower porewater salinities, often <18. A salinity of 18 is considered a threshold above which CH_4_ emissions are negligible as salinity is a proxy for the availability of sulfate supplied from marine water [5]. It may be that the greater density of grass stems in all treatments at Dipper Harbour (Table 4) provided the means to increase emissions of CH_4_ by shunting it from sites of production.

Chronic fertilization resulted in increased N_2_O flux at both marshes, but responses varied with treatment and between the two marshes yet environmental variables were not significant predictors of flux. The varied responses may in part be due to high variability in environmental conditions among plots in each treatment and sampling events. This variability also masks our ability to detect their relationships to fluxes.

Our results indicate that long term nutrient enrichment is likely to affect the global climate impact of the greenhouse gas fluxes of salt marshes, whether microtidal or macrotidal. We used the metrics of SGWP (sustained-flux global warming potential) and SGCP (sustained-flux global cooling potential) for 100 years developed by Neubauer and Megonigal [25] and average annual hourly fluxes weighted over the period marsh soil is not frozen (i.e., excluding December, January, and February) to determine how fertilization treatments changed the climate role of the experimental marsh sites. We see that in the absence of nutrient enrichment (i.e., control plots) both marshes have a negative feedback to global warming, without including soil C sequestration. For both marshes, this negative feedback is driven by the uptake of N_2_O, which (in equivalents of kg CO_2_ m^-2^yr^-1^) is enough to counter the effect of emissions of CH_4_ (Table 6).

**Table 6:** Sustained flux global climate change potential for greenhouse gas fluxes of a micro-tidal and macro-tidal salt marsh with varied chronic fertilization treatments.

	C	N	NPK	high N / low P
Dipper Harbour	average	average	average	average
100 yr SGWP for CH_4_	6.16	6.02	3.65	5.74
100 yr SGWP or SGCP for N_2_O	-6.58	4.48	100.32	20.59
equivalent kg CO_2_ m-^2^yr^-1^	-0.42	10.50	103.97	26.33
contribution of N_2_O to total feedback		0.43	0.96	0.78
difference from Control in N_2_O feedback		10.92	104.39	26.75
Kouchibouguac				
100 yr SGWP or SGCP for CH_4_	1.11	0.74	-5.15	
100 yr SGWP or SGCP for N_2_O	-4.97	17.72	30.37	
equivalent kg CO_2_ m-^2^yr^-1^	-3.85	18.46	25.21	
contribution of N_2_O to total feedback		0.96	1.20	
difference from Control in N_2_O feedback		22.31	29.06	

SGWP = sustained-flux global warming potential, SGCP = sustained-flux global cooling potential.

Nitrogen enrichment, whether as N alone or N available with other nutrients, changed both marshes from N_2_O sinks to sources. Our observations expand on those made by Moseman-Valtierra et al. [4] who reported that their nitrate (only N) applications temporarily converted a salt marsh soil in Massachusetts from a sink to a source. Our study further shows that inclusion of other nutrients, presumably P, increases the emissions of N_2_O over application of N alone. At Dipper Harbour the absence, or muted responses of N_2_O to fertilization with N-alone, or high N/low P suggests to us that P is limiting microbial populations responsible for N transformations, as noted by Sundareshwar and Morris [12]. Thus, over the long term, sources of N pollution without adequate supply of P (e.g., atmospheric deposition) might not result in increased N_2_O emissions from wetlands.

Carbon markets require that C stocks be “permanent” for 100 years, thus we choose this time frame for assessing the change in climate feedbacks due to fertilization in relation to soil C storage. For both marshes, data on net soil C storage is available from previous studies. Connor et al. [26] report rates of C storage from two cores collected from the Dipper Harbour marsh (DHA and DHD) dated with lead-210, which provides rates of soil accumulation over 100 years. Carbon density at 1 cm-intervals was determined from dry bulk density and loss-on-ignition data which had been transformed to C using the relationship reported by Craft et al. [27]. A core from the Kouchibouguac marsh also was dated with lead-210 [28] and its C stock was analyzed in the same manner as the Dipper Harbour cores, but unpublished. The average C stock accumulated over 100 yr is 58.59 g m^-2^ in the two Dipper Harbour cores and 50 g m^-2^ in the Kouchibouguac core. Conversion to rates of CO_2_ sequestration provides a value of 0.2 kg CO_2_ kg m^-2^ yr^-1^ for each site.

With chronic fertilization the global warming potential of the increased N_2_O emissions may be enough to offset the global cooling potential of the C sequestered by salt marshes. The average equivalent CO_2_ sequestration rates calculated for N_2_O and CH_4_ fluxes of fertilized soils are 50 to 500 times higher than the rate of C stored in unfertilized soils. Chronic fertilization also may change the rate of C sequestration of marsh soil, but we are unaware of any reported data to use as a comparison. Fertilization could affect belowground production, a major source of soil carbon, but results of studies have been equivocal, showing both increased and decreased production (e.g., [29–30]). Chronic fertilization could also affect decomposition rates, thus loss of soil C. We expect that autochthonous soil organic matter will have increased N content, making it more labile and subject to more rapid losses due to decomposition. Additional research on the effect of chronic fertilization on soil C storage is needed.

It is difficult to determine to what extent non-experimental, long-term coastal nutrient loading in various regions will have the same impact on N_2_O fluxes as our fertilization treatment. Estimates by Van Drecht et al. [31] of the levels of N and P in urban wastewater around the world indicate that the molar ratio of N to P overall is greater than 11:1. As this is higher than the molar ratio of our high N/low P treatment (8:1), direct exposure of salt marsh soils to urban sewage may result in N_2_O fluxes similar to or less than our experimental results. Vitousek et al. [32] compared nutrient balances in fertilizer applied in a tile-drained corn-soybean rotation in Illinois, USA, a highly fertilized wheat-corn double-cropping system in North China, and a low-input corn-based system in Western Kenya. The fertilizer in North China and Illinois were applied in an N:P molar ratio of 14:1 and 14.7:1, respectively; both considerably higher than the ratio in our high N/low P treatment, suggesting the possibility of a P limitation. With an N:P ratio of only 2:1 the low input fertilization in Western Kenya, ironically, may be more likely to result in increased N_2_O fluxes, based upon our experimental results. However, the balance of N and P received by coastal waters may not be the same as that of the original point and non-point sources. Nitrogen and P loss from fertilized lands depends upon agricultural management. Further, loss of both will depend upon rates of nitirification and denitrification as well as sedimentary processes in rivers (e.g., [33–34]. Thus, additional research on N_2_O fluxes in salt marshes subject to non-experimental fertilization is merited.

## Conclusions

Both marshes have global cooling potential, but this condition changed with fertilization. Our study indicates that coastal eutrophication could cause significant N_2_O fluxes if N is accompanied by an adequate supply of P. Thus, N_2_O fluxes are likely to vary with the source of pollutant nutrients. For instance, increased N loading with atmospheric deposition (NO_3_^-^) may not result in enhanced N_2_O emissions from salt marshes. Previous fertilization experiments performed with applications of N alone may not have resulted in limitation of P due to the short duration of the studies, thus have not shown this effect. We cannot exclude the possibility of increased N from any pollution source affecting CH_4_ fluxes. The shift in soils from a sink to a source of N_2_O reduces the value of the marsh as a blue carbon sink.

## Supporting Information

S1 Appendix Gas flux and environmental data by sample event. (DOCX)
