# Supplementary material for: Greenhouse Gas Fluxes from Salt Marshes Exposed to Chronic Nutrient Enrichment
Source: PLoS One. 2016 Feb 25;11(2):e0149937. doi: 10.1371/journal.pone.0149937 (PMC4767435; doi:10.1371/journal.pone.0149937)
Supplement: S1 Appendix — (DOCX) [file pone.0149937.s001.docx]

| S 1 Appendix. Gas flux and environmental data by sample event. | | | | | | | | | | |
| --- | --- | --- | --- | --- | --- | --- | --- | --- | --- | --- |
| date | plot | CO2 (µmoles/sq m/hr) | CH4 (µmoles/sq m/hr) | N2O (µmoles/sq m/hr) | treatment | marsh | soil temperature (°C) | salinity | water table depth (cm) | # *S. patens* stems per collar |
| April 1, 2012 | KB1 | 1698 | -1.04 | 0.000 | C | Kouchibouguac | 0.5 |  |  | 89 |
| April 1, 2012 | KB2 | 1980 | 1.04 | 0.149 | N | Kouchibouguac | 0.0 |  |  | 114 |
| April 1, 2012 | KB3 | 5426 | 1.94 | 0.149 | NPK | Kouchibouguac | 0.0 |  |  | 364 |
| April 1, 2012 | KB4 | 2947 | 0.45 | 0.000 | C | Kouchibouguac | 1.0 |  |  | 270 |
| April 1, 2012 | KB5 | 5076 | 0.74 | 0.149 | N | Kouchibouguac | 1.0 |  |  | 164 |
| April 1, 2012 | KB6 | 4324 | 0.75 | -0.149 | C | Kouchibouguac | 1.0 |  |  | 45 |
| April 1, 2012 | KB7 | 4061 | -2.23 | 0.000 | NPK | Kouchibouguac | 0.0 |  |  | 424 |
| April 1, 2012 | KB8 | 5733 | 3.42 | 0.000 | C | Kouchibouguac | 0.0 |  |  | 50 |
| April 1, 2012 | KB9 | 3519 | 1.04 | 0.000 | N | Kouchibouguac | 0.0 |  |  | 210 |
| April 1, 2012 | KB10 | 3395 | 1.79 | 0.298 | NPK | Kouchibouguac | 0.0 |  |  | 365 |
| April 1, 2012 | KB11 | 1400 | 0.60 | 0.000 | N | Kouchibouguac | -1.0 |  |  | 200 |
| April 1, 2012 | KB12 | 2805 | 0.15 | 0.000 | NPK | Kouchibouguac | 1.0 |  |  | 90 |
| May 2, 2012 | KB1 | 5058 | -5.04 | -0.146 | C | Kouchibouguac | 4.0 | 15 | 19 | 89 |
| May 2, 2012 | KB2 | 3596 | 0.29 | 0.292 | N | Kouchibouguac | 4.0 | 15 | 5.5 | 114 |
| May 2, 2012 | KB3 | 6491 | 0.00 | 0.292 | NPK | Kouchibouguac | 5.0 | 15 | 15.5 | 364 |
| May 2, 2012 | KB4 | 5661 | -0.88 | -0.147 | C | Kouchibouguac | 5.0 | 11 | 7.9 | 270 |
| May 2, 2012 | KB5 | 9364 | 0.44 | 0.436 | N | Kouchibouguac | 4.0 | 14 | 5.6 | 164 |
| May 2, 2012 | KB6 | 7383 | 1.17 | -0.146 | C | Kouchibouguac | 5.0 | 15 | 2.6 | 45 |
| May 2, 2012 | KB7 | 8706 | 0.73 | 0.145 | NPK | Kouchibouguac | 4.0 | 18 | 4.5 | 424 |
| May 2, 2012 | KB8 | 6206 | 0.65 | 0.000 | C | Kouchibouguac | 3.5 | 11 | 4.1 | 50 |
| May 2, 2012 | KB9 | 3816 | 1.02 | 1.023 | N | Kouchibouguac | 4.0 | 15 | 1.2 | 210 |
| May 2, 2012 | KB10 | 8815 | -0.15 | -0.145 | NPK | Kouchibouguac | 4.0 | 15 | 4.9 | 365 |
| May 2, 2012 | KB11 | 9325 | 1.90 | 1.897 | N | Kouchibouguac | 4.5 | 20 | 2.75 | 200 |
| May 2, 2012 | KB12 | 4109 | 0.00 | 0.000 | NPK | Kouchibouguac | 4.5 | 15 | 5.4 | 90 |
| June 28, 2012 | KB1 | 26284 | -1.42 | -0.142 | C | Kouchibouguac | 14.0 | 12 | 11 | 89 |
| June 28, 2012 | KB2 | 15813 | -0.71 | 0.142 | N | Kouchibouguac | 14.0 | 23 | 0.7 | 114 |
| June 28, 2012 | KB3 | 103713 | 0.28 | 0.993 | NPK | Kouchibouguac | 14.0 | 18 | 0.9 | 364 |
| June 28, 2012 | KB4 | 34492 | -0.43 | -0.143 | C | Kouchibouguac | 14.0 | 16 | 2.7 | 270 |
| June 28, 2012 | KB5 | 88623 | 0.71 | 0.284 | N | Kouchibouguac | 14.0 | 20 | 1 | 164 |
| June 28, 2012 | KB6 | 28367 | 3.13 | -0.142 | C | Kouchibouguac | 14.0 | 19 | -0.5 | 45 |
| June 28, 2012 | KB7 | 60245 | 1.86 | 0.716 | NPK | Kouchibouguac | 15.0 | 20 | -0.7 | 424 |
| June 28, 2012 | KB8 | 42856 | 0.00 | 0.143 | C | Kouchibouguac | 15.0 | 15 | -0.1 | 50 |
| June 28, 2012 | KB9 | 39732 | 0.57 | 0.285 | N | Kouchibouguac | 15.0 | 23 | -0.9 | 210 |
| June 28, 2012 | KB10 | 103116 | -0.14 | 0.286 | NPK | Kouchibouguac | 14.0 | 20 | 1.2 | 365 |
| June 28, 2012 | KB11 | 56689 | -1.00 | 2.277 | N | Kouchibouguac | 14.0 | 20 | -1 | 200 |
| June 28, 2012 | KB12 | 34292 | -1.86 | 2.284 | NPK | Kouchibouguac | 10.0 | 24 | -0.4 | 90 |
| July 4, 2011 | KB1 | 13046 | 0.88 | 0.000 | C | Kouchibouguac | 15.5 | 11 | 12 | 89 |
| July 4, 2011 | KB2 | 11236 | -1.44 | 0.439 | N | Kouchibouguac | 15.0 | 14 | 2 | 114 |
| July 4, 2011 | KB3 | 21442 | -0.31 | 1.870 | NPK | Kouchibouguac | 14.0 | 14 | 3.2 | 364 |
| July 4, 2011 | KB4 | 34696 | 0.19 | -0.312 | C | Kouchibouguac | 14.5 | 10 | 5.75 | 270 |
| July 4, 2011 | KB5 | 52512 | 0.88 | 0.813 | N | Kouchibouguac | 12.9 | 12 | 7 | 164 |
| July 4, 2011 | KB6 | 10369 | 0.56 | 0.000 | C | Kouchibouguac | 16 | 11 | 0.2 | 45 |
| July 4, 2011 | KB7 | 48826 | -2.87 | 0.752 | NPK | Kouchibouguac | 14 | 12 | 0.5 | 424 |
| July 4, 2011 | KB8 | 29035 | 1.56 | -0.375 | C | Kouchibouguac | 14.6 | 10 | 2.5 | 50 |
| July 4, 2011 | KB9 | 16313 | 0.69 | 0.250 | N | Kouchibouguac | 15 | 14 | 0.2 | 210 |
| July 4, 2011 | KB10 | 73417 | 0.38 | 1.442 | NPK | Kouchibouguac | 14 | 11 | 3.1 | 365 |
| July 4, 2011 | KB11 | 31093 | 1.44 | 1.631 | N | Kouchibouguac | 13.5 | 14 | 3.1 | 200 |
| July 4, 2011 | KB12 | 35773 | 0.31 | 1.507 | NPK | Kouchibouguac | 12.9 | 12 | 1.3 | 90 |
| August 8, 2011 | KB1 | 5699 | -0.14 | 0.000 | C | Kouchibouguac | 18.0 | 9 | 34 | 89 |
| August 8, 2011 | KB2 | 8220 | 2.00 | -0.143 | N | Kouchibouguac | 17.0 | 13 | 15.9 | 114 |
| August 8, 2011 | KB3 | 31000 | 2.71 | 0.428 | NPK | Kouchibouguac | 16.0 | 13 | 17.6 | 364 |
| August 8, 2011 | KB4 | 15962 | 0.57 | 0.000 | C | Kouchibouguac | 16.0 | 2 | 34 | 270 |
| August 8, 2011 | KB5 | 23771 | 1.28 | 0.000 | N | Kouchibouguac | 16.0 | 5 | 20 | 164 |
| August 8, 2011 | KB6 | 8586 | 0.21 | 0.000 | C | Kouchibouguac | 16.0 | 10 | 12.7 | 45 |
| August 8, 2011 | KB7 | 37848 | -1.86 | 0.000 | NPK | Kouchibouguac | 15.5 | 7 | 14.8 | 424 |
| August 8, 2011 | KB8 | 17575 | -0.57 | 0.000 | C | Kouchibouguac | 15.5 | 5 | 9.8 | 50 |
| August 8, 2011 | KB9 | 14361 | 0.14 | 0.000 | N | Kouchibouguac | 18.0 | 6 | 2.3 | 210 |
| August 8, 2011 | KB10 | 48920 | 1.71 | 0.000 | NPK | Kouchibouguac | 16.5 | 7 | 15.5 | 365 |
| August 8, 2011 | KB11 | 33761 | 0.14 | 0.000 | N | Kouchibouguac | 17.0 | 11 | 13 | 200 |
| August 8, 2011 | KB12 | 31043 | 0.57 | 1.284 | NPK | Kouchibouguac | 16.0 | 11 | 17 | 90 |
| September 13, 2011 | KB1 | 17501 | 0.71 | -0.142 | C | Kouchibouguac | 14.0 | 10 | 34 | 89 |
| September 13, 2011 | KB2 | 13858 | -0.85 | 0.000 | N | Kouchibouguac | 14.0 | 11 | 15.9 | 114 |
| September 13, 2011 | KB3 | 30912 | -0.28 | 0.424 | NPK | Kouchibouguac | 14.0 | 10 | 17.6 | 364 |
| September 13, 2011 | KB4 | 22527 | -2.12 | 0.283 | C | Kouchibouguac | 14.0 | 9 | 34 | 270 |
| September 13, 2011 | KB5 | 50390 | 0.42 | 0.000 | N | Kouchibouguac | 14.0 | 8 | 20 | 164 |
| September 13, 2011 | KB6 | 10564 | 0.70 | 0.000 | C | Kouchibouguac | 14.0 | 12 | 12.7 | 45 |
| September 13, 2011 | KB7 | 55207 | 0.00 | 1.676 | NPK | Kouchibouguac | 14.0 | 11 | 14.8 | 424 |
| September 13, 2011 | KB8 | 28842 | 0.42 | 0.000 | C | Kouchibouguac | 15.0 | 10 | 9.8 | 50 |
| September 13, 2011 | KB9 | 15560 | -0.14 | 0.000 | N | Kouchibouguac | 16.0 | 11 | 2.3 | 210 |
| September 13, 2011 | KB10 | 56451 | 0.28 | 0.419 | NPK | Kouchibouguac | 13.0 | 11 | 15.5 | 365 |
| September 13, 2011 | KB11 | 23996 | 0.84 | 0.140 | N | Kouchibouguac | 14.0 | 11 | 13 | 200 |
| September 13, 2011 | KB12 | 42014 | 2.10 | 0.000 | NPK | Kouchibouguac | 14.0 | 9 | 17 | 90 |
| October 7, 2011 | KB1 | 2740 | 0.00 | -0.149 | C | Kouchibouguac | 8.0 | 19 | 8.5 | 89 |
| October 7, 2011 | KB2 | 7983 | -3.72 | -0.447 | N | Kouchibouguac | 6 | 22 | -2.5 | 114 |
| October 7, 2011 | KB3 | 7268 | -0.89 | -0.298 | NPK | Kouchibouguac | 6.0 | 22 | -1.1 | 364 |
| October 7, 2011 | KB4 | 7518 | 0.90 | -0.149 | C | Kouchibouguac | 7.0 | 21 | 0 | 270 |
| October 7, 2011 | KB5 | 10040 | 0.45 | 0.000 | N | Kouchibouguac | 7.0 | 21 | 1 | 164 |
| October 7, 2011 | KB6 | 7134 | 0.89 | 0.148 | C | Kouchibouguac | 8.0 | 22 | -1.3 | 45 |
| October 7, 2011 | KB7 | 7074 | -1.49 | 0.297 | NPK | Kouchibouguac | 6.5 | 21 | 0 | 424 |
| October 7, 2011 | KB8 | 17722 | 1.34 | 0.000 | C | Kouchibouguac | 6.5 | 20 | -1.5 | 50 |
| October 7, 2011 | KB9 | 11153 | -1.04 | -0.148 | N | Kouchibouguac | 10.0 | 20 | -2.2 | 210 |
| October 7, 2011 | KB10 | 5295 | -4.15 | 0.000 | NPK | Kouchibouguac | 7.0 | 20 | 0.5 | 365 |
| October 7, 2011 | KB11 | 11569 | -0.30 | 0.149 | N | Kouchibouguac | 7.5 | 23 | -2.5 | 200 |
| October 7, 2011 | KB12 | 14119 | 1.34 | 1.043 | NPK | Kouchibouguac | 6.5 | 20 | -1.1 | 90 |
| November 20, 2011 | KB1 | 1650 | -4.47 | -0.154 | C | Kouchibouguac | 6.0 | 17 | 7 | 89 |
| November 20, 2011 | KB4 | 2067 | 3.86 | 0.154 | C | Kouchibouguac | 4.5 | 16 | 7.5 | 270 |
| November 20, 2011 | KB5 | 6123 | -1.85 | -0.154 | N | Kouchibouguac | 5.5 | 15 | 6.3 | 164 |
| November 20, 2011 | KB6 | 3961 | -0.15 | -0.154 | C | Kouchibouguac | 5.0 | 18 | 2.5 | 45 |
| November 20, 2011 | KB7 | 4827 | -2.62 | 0.000 | NPK | Kouchibouguac | 6.5 | 20 | 2.7 | 424 |
| November 20, 2011 | KB8 | 2067 | 1.39 | 0.154 | C | Kouchibouguac | 7.5 | 19 | 3.7 | 50 |
| November 20, 2011 | KB9 | 4525 | -0.77 | 0.000 | N | Kouchibouguac | 6.0 | 18 | 1 | 210 |
| November 20, 2011 | KB10 | 1188 | -0.31 | 0.154 | NPK | Kouchibouguac | 6.5 | 20 | 3.7 | 365 |
| November 20, 2011 | KB11 | 4997 | 0.77 | 0.154 | N | Kouchibouguac | 5.0 | 20 | 1 | 200 |
| November 20, 2011 | KB12 | 4579 | -2.76 | 0.307 | NPK | Kouchibouguac | 8.5 | 20 | 4.5 | 90 |
| March 20, 2012 | B1 | 2149 | 0.44 | -0.148 | C | Dipper Harbour | 5.0 | 27 | 4 | 169 |
| March 20, 2012 | B2 | 3305 | -0.30 | 0.000 | NPK | Dipper Harbour | 3.0 | 27 | 2.4 | 270 |
| March 20, 2012 | B3 | 2789 | 0.30 | -0.148 | N | Dipper Harbour | 3.5 | 29 | 1.9 | 581 |
| March 20, 2012 | B4 | 4005 | 1.93 | 0.148 | N | Dipper Harbour | 3.5 | 27 | 3 | 452 |
| March 20, 2012 | C1 | 2260 | -0.89 | -0.149 | N | Dipper Harbour | 1.0 | 11 | 4.3 | 491 |
| March 20, 2012 | C2 | 2989 | 2.97 | 0.000 | NPK | Dipper Harbour | 4.0 | 18 | 2.7 | 465 |
| March 20, 2012 | C3 | 2320 | 1.34 | 0.000 | C | Dipper Harbour | 3.0 | 17 | 2.2 | 260 |
| March 20, 2012 | C4 | 4729 | 1.34 | 0.149 | NPK | Dipper Harbour | 2.0 | 20 | 5 | 473 |
| March 20, 2012 | F1 | 3392 | 1.01 | -0.144 | C | Dipper Harbour | 5.0 | 25 | 7.3 | 260 |
| March 20, 2012 | F2 | 5933 | 1.73 | 0.217 | NPK | Dipper Harbour | 5.0 | 21 | 8.5 | 300 |
| March 20, 2012 | H1 | 5038 | 4.04 | 0.144 | C | Dipper Harbour | 4.0 | 19 | 2.5 | 169 |
| March 20, 2012 | H2 | 8834 | 1.15 | 0.144 | N | Dipper Harbour | 2.3 | 21 | 15.5 | 220 |
| March 20, 2012 | Spat F2 | 6991 | -2.03 | -0.145 | high N/low P | Dipper Harbour | 5 | 25 | 14 | 375 |
| March 20, 2012 | Spat F3 | 7543 | -0.29 | 0.145 | high N/low P | Dipper Harbour | 4 | 26 | 13.3 | 227 |
| March 20, 2012 | Spat F5 | 10338 | 1.16 | 0.145 | high N/low P | Dipper Harbour | 3.5 | 28 | 10 | 883 |
| March 20, 2012 | Spat F9 | 3399 | 0.73 | -0.147 | high N/low P | Dipper Harbour | 5 | 17 | 5.1 | 273 |
| March 20, 2012 | SpatC1 | 8022 | -0.43 | -0.145 | C | Dipper Harbour | 5 | 26 | 15 | 358 |
| June 5, 2012 | C3 | 6932 | 7.18 | -0.147 | C | Dipper Harbour | 9.0 | 15 | -0.1 |  |
| June 5, 2012 | C4 | 10661 | 0.00 | 1.319 | NPK | Dipper Harbour | 9.0 | 20 | 0.1 |  |
| June 5, 2012 | F1 | 2692 | -0.29 | 0.146 | C | Dipper Harbour | 10.0 | 30 | -1.6 |  |
| June 5, 2012 | F2 | 4283 | -0.44 | 0.731 | NPK | Dipper Harbour | 9.0 | 24 | 0.5 |  |
| June 5, 2012 | H1 | 6327 | 1.17 | -0.146 | C | Dipper Harbour | 10.0 | 21 | -0.2 |  |
| June 5, 2012 | Spat F9 | 7952 | 0.00 | 0.000 | high N/low P | Dipper Harbour | 9 | 21 |  |  |
| Jun-July 2012 | B1 | 28468 | -1.00 | 0.000 | C | Dipper Harbour | 15 | 33 | 5.6 | 169 |
| Jun-July 2012 | B2 | 51324 | -0.29 | 2.153 | NPK | Dipper Harbour | 14.5 | 29 | 5.5 | 270 |
| Jun-July 2012 | B3 | 26680 | 0.29 | 0.431 | N | Dipper Harbour | 14.5 | 32 | 5.7 | 581 |
| Jun-July 2012 | B4 | 40486 | -0.57 | 0.710 | N | Dipper Harbour | 14 | 34 | 5 | 452 |
| Jun-July 2012 | C1 | 20789 | 9.06 | 0.425 | N | Dipper Harbour | 15 | 10 | 3.9 | 491 |
| Jun-July 2012 | C2 | 41220 | 0.99 | 3.255 | NPK | Dipper Harbour | 15 | 14 | 2.8 | 465 |
| Jun-July 2012 | C3 | 32572 | 11.32 | 0.142 | C | Dipper Harbour | 14.5 | 20 | 2 | 260 |
| Jun-July 2012 | C4 | 71218 | 1.27 | 1.132 | NPK | Dipper Harbour | 14.5 | 24 | 2.4 | 473 |
| Jun-July 2012 | F1 | 15040 | -0.14 | 0.000 | C | Dipper Harbour | 16 | 27 | 0.6 | 260 |
| Jun-July 2012 | F2 | 34614 | -0.28 | 0.707 | NPK | Dipper Harbour | 15 | 32 | 0.7 | 300 |
| Jun-July 2012 | H1 | 24002 | 0.85 | -0.712 | C | Dipper Harbour | 15 | 30 | 5.3 | 169 |
| Jun-July 2012 | H2 | 41220 | -0.71 | 0.000 | N | Dipper Harbour | 15 | 20 | 6.5 | 220 |
| Jun-July 2012 | Spat F2 | 24076 | -2.42 | -0.142 | high N/low P | Dipper Harbour | 15 | 26 | 3.4 | 375 |
| Jun-July 2012 | Spat F3 | 30046 | -0.57 | -0.142 | high N/low P | Dipper Harbour | 15 | 30 | 1.6 | 227 |
| Jun-July 2012 | Spat F5 | 43169 | 0.57 | -0.142 | high N/low P | Dipper Harbour | 14 | 44 | 2.4 | 883 |
| Jun-July 2012 | Spat F9 | 45223 | 4.10 | -0.141 | high N/low P | Dipper Harbour | 15 | 20 | 6.7 | 273 |
| Jun-July 2012 | SpatC1 | 14923 | -0.99 | 0.000 | C | Dipper Harbour | 15 | 30 | 3.8 | 358 |
| July 5, 2011 | B1 | 16532 | 0.96 | 0.000 | C | Dipper Harbour | 14.2 | 30 | 1.7 | 169 |
| July 5, 2011 | B2 | 16879 | 0.00 | 0.386 | NPK | Dipper Harbour | 13 | 25 | 1 | 270 |
| July 5, 2011 | B3 | 18205 | 0.19 | 0.064 | N | Dipper Harbour | 13 | 26 | 1 | 581 |
| July 5, 2011 | B4 | 19905 | 0.13 | 0.193 | N | Dipper Harbour | 12.2 | 24 | 13 | 452 |
| July 5, 2011 | C1 | 13348 | 5.53 | 0.064 | N | Dipper Harbour | 13 | 7 | 10.6 | 491 |
| July 5, 2011 | C2 | 16156 | 9.22 | 0.709 | NPK | Dipper Harbour | 13 | 20 | 0.7 | 465 |
| July 5, 2011 | C3 | 11649 | 12.44 | 0.064 | C | Dipper Harbour | 14 | 10 | 1.3 | 260 |
| July 5, 2011 | C4 | 14479 | -8.96 | 0.387 | NPK | Dipper Harbour | 12.5 | 19 | 0 | 473 |
| July 5, 2011 | F1 | 14765 | -4.71 | -0.387 | C | Dipper Harbour | 13.5 | 21 | 8.5 | 260 |
| July 5, 2011 | F2 | 35392 | -2.26 | 0.645 | NPK | Dipper Harbour | 13.5 | 21 | 0.3 | 300 |
| July 5, 2011 | Spat F2 | 18640 | -2.26 | 2.514 | high N/low P | Dipper Harbour | 14.8 | 24 | 0 | 375 |
| July 5, 2011 | Spat F3 | 14817 | 3.48 | 2.579 | high N/low P | Dipper Harbour | 14 | 26 | 1.4 | 227 |
| July 5, 2011 | Spat F5 | 49112 | 2.45 | 2.772 | high N/low P | Dipper Harbour | 15.3 | 24 | 3.9 | 883 |
| July 5, 2011 | SpatC1 | 9557 | 2.90 | -0.064 | C | Dipper Harbour | 14 | 21 | 1.2 | 358 |
| August 9, 2011 | B1 | 48161 | 0.87 | 0.000 | C | Dipper Harbour | 15.5 | 32 | 0.7 | 169 |
| August 9, 2011 | B2 | 18143 | 0.87 | 0.000 | NPK | Dipper Harbour | 15.5 | 32 | 2.5 | 270 |
| August 9, 2011 | B3 | 18910 | 1.73 | 0.000 | N | Dipper Harbour | 15.5 | 31 | 2.2 | 581 |
| August 9, 2011 | B4 | 19231 | 1.30 | 0.000 | N | Dipper Harbour | 16 | 30 | 7 | 452 |
| August 9, 2011 | C1 | 8219 | 1.88 | 0.000 | N | Dipper Harbour | 16 | 22 | 1 | 491 |
| August 9, 2011 | C2 | 11003 | 1.74 | -0.145 | NPK | Dipper Harbour | 16 | 16 | 3 | 465 |
| August 9, 2011 | C3 | 7959 | -2.39 | -0.217 | C | Dipper Harbour | 15 | 20 | 1 | 260 |
| August 9, 2011 | C4 | 14758 | 0.29 | 0.000 | NPK | Dipper Harbour | 15 | 25 | 2.6 | 473 |
| August 9, 2011 | F1 | 22913 | 0.29 | 0.000 | C | Dipper Harbour | 15.5 | 28 | 18 | 260 |
| August 9, 2011 | F2 | 27511 | -0.72 | 0.000 | NPK | Dipper Harbour | 18 | 25 | 5 | 300 |
| August 9, 2011 | H1 | 18454 | -0.72 | 0.000 | C | Dipper Harbour | 16.5 | 24 | 8 | 169 |
| August 9, 2011 | Spat F2 | 28634 | 5.05 | 0.000 | high N/low P | Dipper Harbour | 16 | 31 | 2.1 | 375 |
| August 9, 2011 | Spat F3 | 26528 | 0.00 | 0.000 | high N/low P | Dipper Harbour | 16 | 30 | 1.9 | 227 |
| August 9, 2011 | Spat F5 | 71756 | 0.00 | 0.144 | high N/low P | Dipper Harbour | 16 | 30 | 4 | 883 |
| August 9, 2011 | Spat F9 | 27368 | 2.31 | 0.433 | high N/low P | Dipper Harbour | 15 |  | 8.4 | 273 |
| August 9, 2011 | SpatC1 | 13790 | -0.72 | 0.144 | C | Dipper Harbour | 15 | 30 | 3 | 358 |
| September 12, 2011 | B1 | 14379 | 11.66 | 0.000 | C | Dipper Harbour | 14.3 | 26 | 1.2 | 169 |
| September 12, 2011 | B2 | 12650 | -0.43 | 4.340 | NPK | Dipper Harbour | 13.0 | 30 | 1.5 | 270 |
| September 12, 2011 | B3 | 12368 | -1.74 | 0.000 | N | Dipper Harbour | 11.0 | 30 | 2.6 | 581 |
| September 12, 2011 | B4 | 16007 | 1.44 | 0.144 | N | Dipper Harbour | 11.0 | 29 | 2.2 | 452 |
| September 12, 2011 | C1 | 9588 | 7.92 | 0.000 | N | Dipper Harbour | 16.0 | 11 | 8.2 | 491 |
| September 12, 2011 | C2 | 9564 | 3.16 | 6.319 | NPK | Dipper Harbour | 13.0 | 20 | 1.5 | 465 |
| September 12, 2011 | C3 | 9588 | -1.15 | -0.217 | C | Dipper Harbour | 15.5 | 18 | 3.6 | 260 |
| September 12, 2011 | C4 | 10812 | 1.30 | 0.000 | NPK | Dipper Harbour | 14.0 | 24 | 2 | 473 |
| September 12, 2011 | F1 | 10785 | 0.00 | 0.000 | C | Dipper Harbour | 14.5 | 27 | 7.5 | 260 |
| September 12, 2011 | F2 | 16672 | 3.02 | 10.078 | NPK | Dipper Harbour | 15.0 | 26 | 9.2 | 300 |
| September 12, 2011 | H1 | 16583 | -0.43 | 0.000 | C | Dipper Harbour | 15.0 | 22 | 10.7 | 169 |
| September 12, 2011 | H2 | 33707 | -0.43 | 0.000 | N | Dipper Harbour | 14.0 | 25 | 6.6 | 220 |
| September 12, 2011 | Spat F2 | 14595 | -0.43 | 0.144 | high N/low P | Dipper Harbour | 14.0 | 26 | 3.1 | 375 |
| September 12, 2011 | Spat F3 | 12648 | 0.58 | 0.433 | high N/low P | Dipper Harbour | 14.0 | 28 | 2.3 | 227 |
| September 12, 2011 | Spat F5 | 21993 | 1.87 | 0.000 | high N/low P | Dipper Harbour | 13.5 | 27 | 4 | 883 |
| September 12, 2011 | Spat F9 | 11778 | 2.61 | 0.000 | high N/low P | Dipper Harbour | 14.5 | 29 | 7 | 273 |
| September 12, 2011 | SpatC1 | 8451 | -0.43 | 0.144 | C | Dipper Harbour | 14.0 | 27 | 3.6 | 358 |
| October 8, 2011 | B1 | 14002 | -0.43 | -0.285 | C | Dipper Harbour | 12.0 | 27 | 8 | 169 |
| October 8, 2011 | B2 | 26606 | -1.28 | 3.140 | NPK | Dipper Harbour | 10.0 | 29 | 10 | 270 |
| October 8, 2011 | B3 | 28361 | 0.86 | 0.286 | N | Dipper Harbour | 10.0 | 30 | 12.1 | 581 |
| October 8, 2011 | B4 | 24316 | -0.57 | -0.286 | N | Dipper Harbour | 12.5 | 29 | 10.7 | 452 |
| October 8, 2011 | C1 | 7208 | 0.14 | 0.143 | N | Dipper Harbour | 10.0 | 11 | 6.8 | 491 |
| October 8, 2011 | C2 | 22466 | 1.28 | 4.139 | NPK | Dipper Harbour | 8.0 | 21 | 6.8 | 465 |
| October 8, 2011 | C3 | 8093 | 1.86 | 0.000 | C | Dipper Harbour | 9.5 | 15 | 3 | 260 |
| October 8, 2011 | C4 | 21938 | 0.14 | 5.138 | NPK | Dipper Harbour | 9.0 | 22 | 6 | 473 |
| October 8, 2011 | F1 | 4635 | 0.72 | -0.286 | C | Dipper Harbour | 9.5 | 25 | 8.1 | 260 |
| October 8, 2011 | F2 | 14592 | 0.14 | 4.006 | NPK | Dipper Harbour | 11.0 | 25 | 15 | 300 |
| October 8, 2011 | H1 | 8135 | 1.58 | -0.143 | C | Dipper Harbour | 11.5 | 22 | 12 | 169 |
| October 8, 2011 | H2 | 16629 | -0.29 | 0.000 | N | Dipper Harbour | 9.0 | 26 | 15.8 | 220 |
| October 8, 2011 | Spat F2 | 13463 | 4.58 | -0.143 | high N/low P | Dipper Harbour | 7.5 | 28 | 10.8 | 375 |
| October 8, 2011 | Spat F3 | 15268 | 1.72 | -0.286 | high N/low P | Dipper Harbour | 10.0 | 27 | 12.5 | 227 |
| October 8, 2011 | Spat F5 | 25380 | 1.29 | 0.143 | high N/low P | Dipper Harbour | 8.5 | 27 | 7.5 | 883 |
| October 8, 2011 | Spat F9 | 16300 | 1.72 | -0.572 | high N/low P | Dipper Harbour | 9.5 | 21 | 12.2 | 273 |
| October 8, 2011 | SpatC1 | 12203 | -0.86 | 0.000 | C | Dipper Harbour | 8.5 | 29 | 11.2 | 358 |
| November 19, 2011 | B1 | 2276 | 4.43 | 0.148 | C | Dipper Harbour | 5.0 | 23 | 5.7 | 169 |
| November 19, 2011 | B2 | 2749 | 1.33 | 0.000 | NPK | Dipper Harbour | 4.0 | 25 | 5 | 270 |
| November 19, 2011 | B3 | 1744 | 1.18 | 0.000 | N | Dipper Harbour | 5.0 | 25 | 5 | 581 |
| November 19, 2011 | B4 | 2438 | 0.59 | -0.148 | N | Dipper Harbour | 5.0 | 25 | 3.8 | 452 |
| November 19, 2011 | C1 | 626 | 2.36 | 0.000 | N | Dipper Harbour | 5.5 | 10 | 5.2 | 491 |
| November 19, 2011 | C2 | 2497 | 1.77 | 0.148 | NPK | Dipper Harbour | 5.5 | 16 | 2.8 | 465 |
| November 19, 2011 | C3 | 1433 | 3.84 | 0.000 | C | Dipper Harbour | 5.5 | 14 | 2.2 | 260 |
| November 19, 2011 | C4 | 2320 | 3.40 | 0.296 | NPK | Dipper Harbour | 5.5 | 20 | 2.8 | 473 |
| November 19, 2011 | F1 | 1773 | 0.15 | -0.147 | C | Dipper Harbour | 5.0 | 20 | 4.6 | 260 |
| November 19, 2011 | F2 | 2593 | 0.00 | 0.440 | NPK | Dipper Harbour | 5.5 | 22 | 7.1 | 300 |
| November 19, 2011 | H1 | 1802 | -0.84 | -0.065 | C | Dipper Harbour | 4.5 | 19 | 9.8 | 169 |
| November 19, 2011 | H2 | 2879 | -1.03 | 0.000 | N | Dipper Harbour | 5.0 | 20 | 13.7 | 220 |
| November 19, 2011 | Spat F2 | 3225 | 0.59 | 0.000 | high N/low P | Dipper Harbour | 7.0 | 27 | 8 | 375 |
| November 19, 2011 | Spat F3 | 4295 | 0.00 | -0.147 | high N/low P | Dipper Harbour | 9.5 | 28 | 9.7 | 227 |
| November 19, 2011 | Spat F5 | 4310 | 3.96 | 0.000 | high N/low P | Dipper Harbour | 7.0 | 27 | 4.2 | 883 |
| November 19, 2011 | Spat F9 | 3460 | 1.76 | -0.293 | high N/low P | Dipper Harbour | 6.0 | 26 | 11 | 273 |
| November 19, 2011 | SpatC1 | 2917 | -3.08 | 0.000 | C | Dipper Harbour | 8.0 | 27 | 7.8 | 358 |
